# Integrated Evaluation of Reliability and Power Consumption of Wireless Sensor Networks

**DOI:** 10.3390/s17112547

**Published:** 2017-11-05

**Authors:** Antônio Dâmaso, Nelson Rosa, Paulo Maciel

**Affiliations:** Centro de Informática, Universidade Federal de Pernambuco, Av. Jornalista Aníbal Fernandes, s/n-Cidade Universitária, 50740-560 Recife, PE, Brazil; avld@cin.ufpe.br (A.D.); prmm@cin.ufpe.br (P.M.)

**Keywords:** power consumption, reliability, wireless sensor networks, sensitivity analysis, formal models

## Abstract

Power consumption is a primary interest in Wireless Sensor Networks (WSNs), and a large number of strategies have been proposed to evaluate it. However, those approaches usually neither consider reliability issues nor the power consumption of applications executing in the network. A central concern is the lack of consolidated solutions that enable us to evaluate the power consumption of applications and the network stack also considering their reliabilities. To solve this problem, we introduce a fully automatic solution to design power consumption aware WSN applications and communication protocols. The solution presented in this paper comprises a methodology to evaluate the power consumption based on the integration of formal models, a set of power consumption and reliability models, a sensitivity analysis strategy to select WSN configurations and a toolbox named EDEN to fully support the proposed methodology. This solution allows accurately estimating the power consumption of WSN applications and the network stack in an automated way.

## 1. Introduction

Sensor Wireless Networks (WSNs) are a kind of ad hoc network made up of hundreds or thousands sensor nodes that cooperate to route packets to a particular node (sink node) capable of mediating the communication between the WSN and an external network, e.g., the Internet. Commonly deployed in environments whose access is hard, sensor nodes have limited resources of processing, storage, transmission, and battery. In such places, it is painful to replace a sensor node when it fails or when the battery is over. Therefore, to plan the WSN deployment is a crucial activity.

An initial step to planning a WSN is to assess the power consumption of application and communication protocols running on the sensor nodes. By analysing this assessment, it is possible to infer the WSN lifetime. However, this is not sufficient as the WSN can have the energy to perform for days, but some nodes may be unable to communicate with the sink node. In other words, some portions of the network can be dead in the case the batteries of some nodes are drained. As existing solutions [1,2,3,4] usually calculate the reliability of WSNs considering the failure of communication links and nodes, if a node fails (stops working) due to the scarcity of energy, the WSN’s reliability is affected by the node’s power consumption. An approach to face this problem is to evaluate the WSN’s reliability integrated with the power consumption. If this evaluation is ignored, the WSN could not perform as planned.

A strategy to estimate both the reliability and power consumption of WSNs is to use actual sensor nodes [1]. This solution, however, is harmful because it requires several sensors, e.g., to evaluate a WSN with thousand of nodes. Besides, it is also limited as practical scenarios are difficult to be replicated. Another alternative is to use simulators or formal models. The traditional simulators used to assess the WSN power consumption can also evaluate its reliability. Some works like [2,3] consider the percentage of packet loss or corrupted to calculate the WSN reliability. However, these works consider simply the failure of communication links and neglect that the node can also fail. Other works, like [4], propose a simulator and a formal model to assess reliability and WSN power consumption. In this case, it considers the failures of both sensor nodes and communication links. However, they do not consider the power consumption of the application running on the node.

By observing the importance of planning WSNs and the problems aforementioned, this paper presents a solution to estimate the power consumption of WSN infrastructure and applications using models and taking in account their reliabilities. The proposal consists of three key elements: a methodology to guide the integrated evaluation, a set of CPN (Coloured Petri Net) [5,6,7] and RBD (Reliability Block Diagram) [8] models, a sensitivity analysis-based strategy to select WSN configurations, and a tooling that automatises the steps of the methodology. The methodology guides all steps to plan a WSN considering its power consumption and reliability and uses the models to express both characteristics. Furthermore, a set of tools fully supports the adoption of the methodology. For example, the tooling helps users to improve the power consumption and reliability of WSNs by giving suggestions or evaluating several WSN configurations simultaneously using the sensitivity analysis [9].

This work has the following unique contributions: a methodology that guides the integrated evaluation of power consumption and reliability of WSNs; a tooling that supports the methodology and automatizes the whole assessment process; formal models to represent reliability and power consumption the WSN applications and network stack; and the sensitivity analysis-based strategy to help in selecting WSN configurations.

This document is organised as follows. Section 2 presents basic concepts necessary in the remaining of this paper. Next, Section 3 presents the proposed methodology. Section 4 describes the proposed basic power consumption and reliability models and how to compose them. Section 5 describes the tooling that supports the methodology. Section 6 shows the experimental evaluation carried to validate the proposed models. Current researchers associated with the analysis of reliability and power consumption of WSNs are introduced in Section 7. Lastly, Section 8 presents the final remarks and some directions for future work.

## 2. Background

This section introduces basic concepts of CPN (Coloured Petri Net) [5,6,7] and RBD (Reliability Block Diagram) [8] used to model the WSN power consumption and reliability, respectively. Meanwhile, it also introduces the concept of sensitivity analysis.

### 2.1. Coloured Petri Net

Petri nets (PN) [10] is a class of formalisms used to model an extensive variety of systems by a graphical notation. Petri nets enable us to check properties such as reachability, boundedness, liveness and reversibility [11]. Currently, PNs have several extensions, such as timed, stochastic, and coloured. Whatever the extension, however, it has four primary elements: places to express system variables, transitions to model actions (or events) occurring in the system, arcs to indicate relations between places and transitions and tokens that are places’ values.

**Figure 1:**
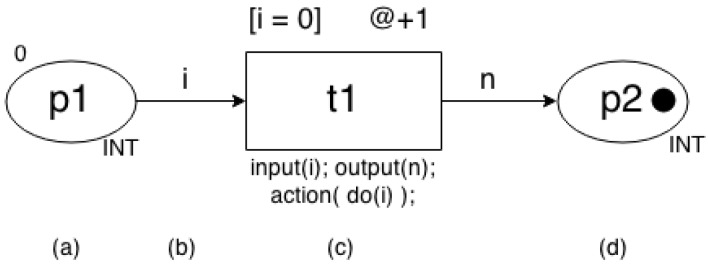
(**a**) place, (**b**) arc and (**c**) transition and (**d**) place with token representations in CPN Tools.

Coloured Petri net (CPN) [5,6,7] is a PN extension that combines high-level programming language capabilities with core elements of Petri nets. CPN Tools [12] is the first tooling adopted to create coloured Petri nets. In CPN, every place has associated three basic information: a name to identify the place, a colour set to define the type of token stored in the place, and an initial marking whose objective is to start the CPN model. Figure 1a shows a place (*p*1) storing one token whose value is set to 0. Outside the place, it is possible to see an initial marking (set to 0) and a colour set (INT).

Figure 1b depicts an arc annotated with a variable *i* whose type is the same as one of the input place’s colour. Figure 1c represents a transition including: identification (*t*1), guard, time and code. Guard ([i = 0]) is a logical expression to enable/disable the transition. When the *t*1 fires, time fragment (@ + 1) computes a specific amount of time and the code associated to the transition is executed. The code include an input, an output and an action. The action has the programming code to processes the input (input) and returns a value (output).

Hierarchical CPN enables us to design CPN models possibly connected to each other and at different abstraction levels. This characteristic is helpful for building large models, such as ones to describe an application or a protocol stack. It is worth observing that this feature allows dividing the model into various modules where each one has a CPN.

### 2.2. Reliability Block Diagram

Reliability Block Diagram (RBD) [13] is widely used to model and evaluate the reliability of systems [8]. RBD allows both representing and estimating the reliability of a system through combining a collection of blocks, where each block expresses the reliability of a given element of the system, e.g., the reliability of a switch.

**Figure 2:**
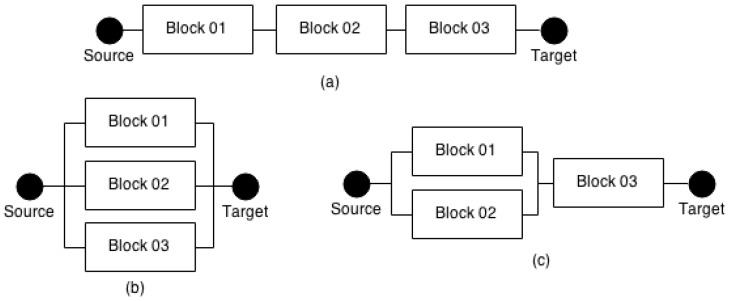
RBD organized in (**a**) series, (**b**) parallel and (**c**) combined.

As illustrated in Figure 2, an RBD has an input (*Source*) and an output (*Target*) that model the beginning and the ending of the system, respectively. Blocks are organised in series, parallel or their combinations. Furthermore, they have only two states, namely *failure* or *working*. Failures are independent of each other, and each block has a reliability associated. For example, block $B_{i}$ has reliability $R_{i}\left(t\right)$ associated to "working state" at time *t*. Meanwhile, $1-R_{i}\left(t\right)$ represents the “failure state” at time *t*.

In the case a system works properly when its components are operational, the corresponding RBD is structured as a series of system’s components. By considering a given series structure composed of *n* independent components, as presented in Figure 2a, the system reliability is calculated using Equation (1).

(1) $$R\left(t\right)=\prod_{i=1}^{n}R_{i}\left(t\right)$$

$R_{i}\left(t\right)$ is the reliability of block $b_{i}$ at time *t*. Consequently, the reliability at time *t* of a system having *n* components in series is calculated by the product of the reliability of the blocks that compose it. For instance, an RBD having three blocks has reliability equal to 0.729, if the reliability of each one is 0.9.

On the other hand, when a given system, made up of *n* components, only works if at least one of them operates correctly, the corresponding RBD is structured in parallel. In this way, the reliability at time *t* of the system shown in Figure 2b is computed by Equation (2).

(2) $$R\left(t\right)=1-\prod_{i=1}^{n}\left(1-R_{i}\left(t\right)\right)$$

In this case, given a system having three blocks (each one having its reliability set to 0.9), its reliability is 0.999. It is worth observing how the organisation of the blocks affects the reliability of the entire system.

In addition to series and parallel combinations, additional combinations can be created, such as series-parallel, parallel-series, bridge and *k-out-of-n*. The reliability of these additional combinations also use Equations (1) and (2). Figure 2c shows a RBD series-parallel where *Block01* and *Block02* are in parallel, and *Block03* is in series. In this case, the reliability of parallel structure is calculated first, and then the series one. Assuming that each block has reliability equal to 0.9, the reliability of the whole system is 0.891.

### 2.3. Sensitivity Analysis

A system can be described as a black box that receives a set of inputs (parameters) and generates a set of outputs (results). When we change the parameters, the system can produce new results or not. Observing this, the sensitivity analysis checks which parameters (inputs) interfere in the results (output) of the system [9].

An uncomplicated way to perform the sensitivity analysis is changing one parameter at a time while keeping the other constant. Therefore, it is possible to compare the results according to the modified parameter. Besides that, experiment design techniques (Design of Experiments—DoE) can be used to determine (simultaneously) the different effects and interaction between various parameters that can affect the output [14]. In DoE, parameters and their values are referred as factors and levels, respectively. To experiment using DoE techniques, it is necessary to define the factors and their levels, create and evaluate all possible combinations. The number of combinations (*C*) is calculated as follows [14]:

(3) $$C=\prod_{i=1}^{n}level(f_{i})$$

where *n* is the total number of factors, and *level($f_{i}$)* is a function that returns the amount of levels of factor $f_{i}$. Depending on the number of factors and levels, the sensitivity analysis may require a lot of processing and time, which can derail the study. An alternative is to limit the number of factors and/or levels.

We can do a sensitivity analysis with only two levels and with *k* factors. This type of experiment is called $2^{k}$ design and the number of combinations is equal to $2^{k}$. Nevertheless, depending on the number of factors, the number of combinations can be large. After obtaining the results, we can create a ranking of the factors and the interactions between them that influenced the results. Therefore, it is necessary to calculate the impact (*S*) of one factor (θ) in a particular output (*Y*). The effect of factor θ is the difference between the averages of the first ($\theta_{-1}$) and the second levels ($\theta_{+1}$), as shown in Equation (4).

(4) $$S_{a}\left(Y\right)=T_{Y}\left(\theta_{+1}\right)-T_{Y}\left(\theta_{-1}\right),$$

where *T* is a function that returns output *Y* of the average obtained with the first level ($\theta_{-1}$) and the second level ($\theta_{+1}$) of factor θ. Besides that, we must use this equation to calculate the interaction between two factors. For example, the interaction between factors *A* and *B* is calculated as follows:

(5) $$S_{a}\left(Y\right)=\frac{T_{Y}\left(A_{+1},B_{+1}\right)+T_{Y}\left(A_{-1},B_{-1}\right)}{2}-\frac{T_{Y}\left(A_{+1},B_{-1}\right)+T_{Y}\left(A_{-1},B_{+1}\right)}{2}$$

where function $T_{Y}\left(A_{+1},B_{+1}\right)$ returns the average of the output *Y* when the value of the factors *A* and *B* is the second level; function $T_{Y}\left(A_{-1},B_{-1}\right)$, when the value of the factors *A* and *B* is the first level; and so on.

## 3. Methodology

This section presents the proposed methodology to design WSN infrastructures and applications considering their power consumption and reliability. As mentioned in Section 1, the proposed methodology integrates reliability into the power consumption evaluation, and it is based on the generation and simulation of formal models described in CPN and RBD as shown in Section 4.

**Figure 3:**
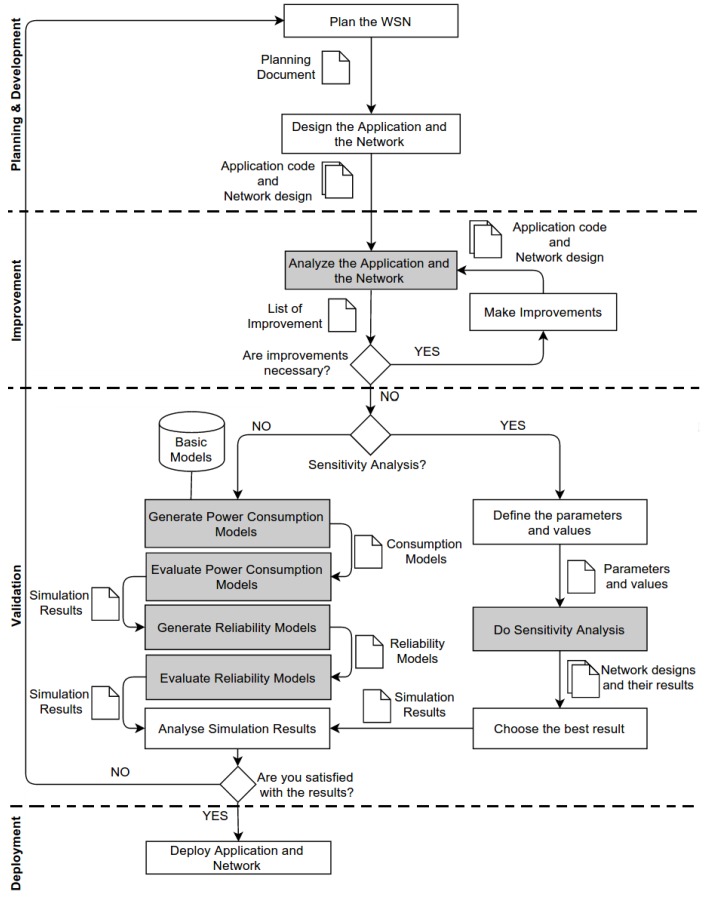
Proposed methodology.

The methodology presented in Figure 3 is divided into four phases: *Planning and Development*, *Improvement*, *Validation* and *Deployment*. It includes a set of manual (white rectangles) and semi-automatic activities (grey rectangles), their connections (arrows), generated artefacts, decision points (diamonds) and a repository. A manual activity is one performed exclusively by the user, and a semi-automatic activity is primarily carried out by a tool and demands either no action or small intervention of the user. Decision points allow the user or a tool to select a particular flow of the methodology. Artefacts are generated as a result of an activity and serve as input to the next activity. Finally, the repository stores reusable models used to specify the power consumption.

**Planning and Development.** In this phase, the user defines the network and application conditions, and create the WSN based on these requirements. Hence, the first activity of the methodology, namely *Plan the WSN*, is a manual action whose objective is to define functional and non-functional requirements of the WSN and WSN applications. For example, the user should specify the operating system and programming language used to implement the WSN applications. He/she can also determine what kind of data the sensor node collects, WSN regions and lifetime, WSN deployment area, environment conditions (e.g., interference and noise), routing protocols and the number of sensor nodes. This information is put in *Planning Document*, which must be used for the next activity to create the WSN.

Activity *Design the Application and Network* creates the WSN based on *Planning Document*. The user manually implements the application code to be installed in the sensor node and creates the network design (e.g., WiseML [15]) that configures and deploys virtual sensor nodes at a virtual environment like in the real situation. These two artefacts (application code and network design) are based on the WSN planning and will be used to validate the WSN.

**Improvement**. In this phase, the user must analyse the WSN to find inconsistencies among the requirements or to improve the power consumption and reliability of the WSN, based on recommendations to all WSNs. Some inconsistencies can be found, and standards recommendations can be suggested only by analysing the features of the application code and the network design without their evaluation. This phase can be semi-automatic because some inconsistencies and recommendations are already known and common to WSNs.

For example, if a sensor node has not a direct access to the sink node, protocols DIRECT and LEACH are not appropriate for this WSN, requiring choose another routing protocol or change the WSN configuration. This kind of check (e.g., if the distance between the sensor node and the sink node is less than or equal to the communication radius of the sensor node) can be performed without the CPN and RBD models. Regarding the application, an improvement could be to optimise the application code to reduce the power consumption of the sensor node. For example, the application should avoid parentheses or should use a constant instead of a variable when the value does not change. These patterns can be automatically analysed and suggested to the user, which must decide to apply or not the suggestion (activity *Make Improvements*).

Furthermore, it is also important to guarantee that the proposed improvements do not conflict with the functional requirements defined in the *Planning Document*. For instance, the application behaviour does not change, the sensor node collects the same information (e.g., temperature), the topology is the same (e.g., hierarchical), among others.

**Validation.** It is necessary to validate the WSN before installing it on the field. This methodology uses the evaluation of the power consumption and reliability of the WSN as part of the validation process. The user may decide to evaluate one or a set of WSNs. If he/she wants to evaluate the power consumption and reliability of a single WSN, activity *Generate Power Consumption Models* is automatic and yields CPN models that express the power consumption of the application (Section 4.1), network (Section 4.2) and sensor node (Section 4.3). These models are also evaluated (activity *Evaluate Power Consumption Models*) to estimate the power consumption, and whose results are used in the next activity, namely *Generate Reliability Models*.

Similarly to the previous activity, activity *Generate Reliability Models* is automatic and creates RBD models (Section 4.4) used to evaluate the WSN reliability. These models are created using information from the network design (e.g., WSN regions, sensor nodes and routing algorithm) and the reliability of the sensor nodes based on the power consumption results. After the RBD models are evaluated (activity *Evaluate Reliability Models*), all simulation results (power consumption and reliability) are presented to the user.

Sometimes, it is necessary to evaluate the WSN with different parameters to see which WSN configuration satisfies what was planned. For example, communication protocol A has a power consumption better than communication Protocol B? The WSN reliability improves if the region A has ten sensor nodes instead of five? He/she must set the parameters (e.g., routing protocol) and their values (e.g., DIRECT and LEACH) in activity *Define the parameters and values*. The next activity (*Do Sensitivity Analysis*) automatically creates and evaluates the power consumption and reliability of all WSN configurations. After evaluating all WSNs created, it generates all WSNs, their respective results and impact of each parameter. The user must choose the WSN configuration that has the best results according to the criteria defined in the *Planning Document*.

Finally, the user analyses the results produced by the evaluation process (*Analyse Simulation Results*). For example, he/she needs to verify if the WSN lifetime, WSN behaviour, the reliability of the regions, throughput and others information produced by the evaluation process are according to the WSN requirements defined in *Planning Document*. Then, the user decides to deploy the WSN in the field or repeats the whole process (phase *Planning and Development*).

**Deployment.** The last phase consists of deploying the WSN in the area as planned (activity *Deploy Application and Network*). If the user deploys a WSN with characteristics (e.g., the number of sensor nodes, the number of sink nodes, or routing protocols) different from what was planned, the WSN will not perform as it was designed, e.g., the WSN can consume more energy than expected. Thus, a new WSN planning must be done and evaluated.

## 4. Models

This work defined four set of models to estimate the power consumption and the reliability of WSNs. These models are used in several activities of the methodology introduced in the previous section. Due to the complexity of evaluating the power consumption, it was necessary to define three different models to evaluate the power consumption: Consumption Application Model (nesC application); Consumption Network Model (network); and Consumption Sensor Node Model (combined power consumption of application and network). The reliability model, named Reliability Model, is used to assess the reliability of a WSN region. Next subsections present all these four models.

### 4.1. Consumption Application Model

Consumption Application Models [16] are used in activity *Generate Power Consumption Models* of the proposed methodology (see Figure 3). These model are described in CPN and used to evaluate the power consumption of WSN applications implemented in nesC. To perform this task, it is necessary (i) to identify the nesC operators used in the application and convert them into Operator Models; (ii) map the nesC functions into Function Models; and, finally, (iii) create the Application Model, by joining all Functions Models with the Scheduler Model. In this way, different WSN applications have different models, and (consequently) different power consumptions.

**Figure 4:**
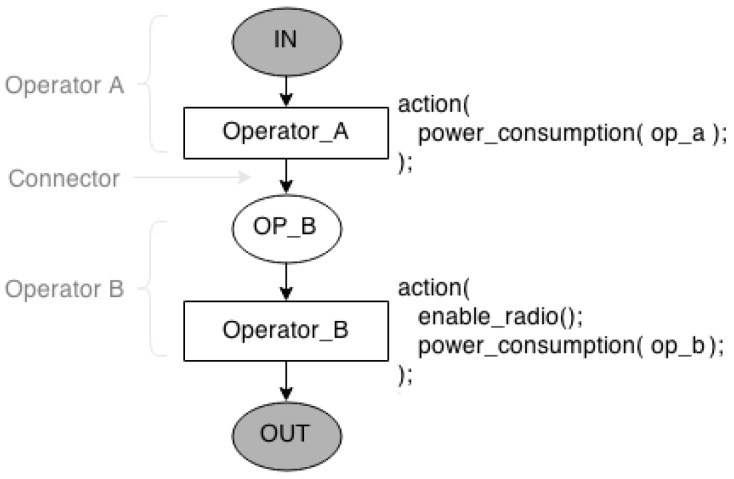
Generic Consumption Function Model.

An Consumption Operator Model is a CPN model that represents the power consumption of a nesC operator. Figure 4 shows two Operator Models and each model has one place and one transition. For example, the operator model of *Operator A* is represented by place *IN* and transition *Operator_A*, while *Operator B* is modelled as place *OP_B* and transition *Operator_B*. When transition *Operator_A* is triggered, it records the power consumption of the operator being modeled (*power_consumption(op_a)*). Beside that, it can indicate whether a feature is enabled/disabled (such as the radio) or if another implemented function must be performed. For example, the transition *Operator_B* enables the radio.

NesC loop statements (*for*, *while* and *do-while*) and selection statements (*if-then-else* and *switch-case*) have a operation models more complex to represent their behaviours as introduced in [16].

As shown in Figure 4, operator models together compose a Consumption Function Model, which represents a function implemented in the application. The number of operator models used to describe a function model depends on the number of operators used in the function being modelled. For example, if a function has two operators (*Operator_A* and *Operator_B*), its function model has two operator models connected by an arc (*Connector*). The function model starts and ends with places representing the beginning (*IN*) and the ending (*OUT*) of the function being modelled.

**Figure 5:**
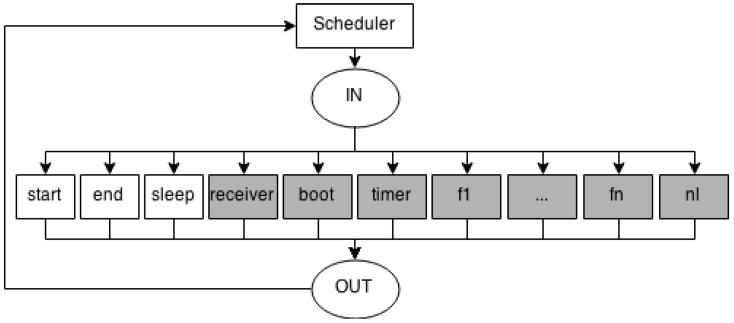
Consumption Scheduler Model.

After modelling all functions of the application, it is necessary to create a single model that orders their execution. This is the responsibility of the Consumption Scheduler Model (see Figure 5). This model has one transition for each Consumption Function Model created (*f1*, *f2*, *f3*, ..., *fn*); one transition called *Scheduler* for managing the execution of the Function models; and three transitions to represent the states of the sensor node (*start*, *sleep* and *end*).

### 4.2. Consumption Network Model

The Consumption Network Model is responsible for evaluating the power consumption of the network [16] as defined in activity *Evaluate Power Consumption Models* (see Figure 3). This model is defined by composing three models: two models that express the power consumption of the network and link layers (protocol stack); and an Environment Model, which is responsible for forwarding the packets among the sensors nodes.

**Figure 6:**
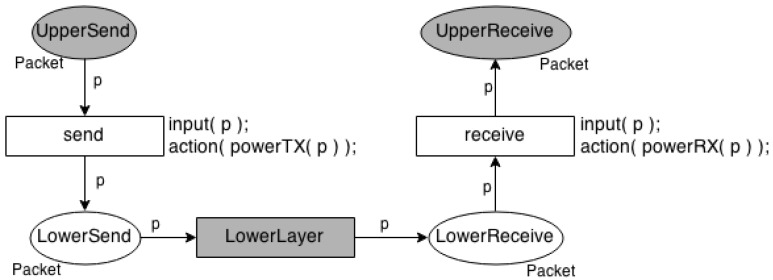
Template of protocol models.

Due to the complexity of creating a generic model to express the power consumption of different WSN communication protocols, a template was proposed instead (see Figure 6). This template includes basic elements used to specify several communication protocols. It defines that any protocol model should contain two places (*UpperSend* and *UpperReceive*) to interact with the upper layer and a transition *LowerLayer* that acts as a bridge to the lower layer.

For example, in a network layer protocol, the places *UpperSend* and *UpperReceive* are connected to the upper layer via places *in* and *out* in the Consumption Scheduler Model (Figure 5), respectively; and a transition (*LowerLayer*) represents the lower layer connecting the places *LowerSend* and *LowerReceive* with the places *UpperSend* and *UpperReceive* in the link layer model, respectively. We should put transitions and places between place *UpperSend* and transition *LowerLayer* to represent actions to send a packet. Additionally, we can put transitions and places between place *UpperReceive* and transition *LowerLayer* to represent actions to receive a packet.

In this paper, we use the energy math model named *First Order Radio Model* [17]. This model calculates the power consumption of the network and has been widely adopted in WSNs. The model calculates the power consumption for sending and receiving packets. In practice, the power consumption of the packet processing is ignored because the sensor node consumes more power in packet transmission than in its processing.

Due to these characteristics, the power consumption of the Network model is calculated in the link layer, because it sends and receives packets to/from the environment. For example, the template proposed in Figure 6 shows two functions, namely *powerTX*() and *powerRX*(), to calculate the power consumption for sending and receiving packets, as defined in *First Order Radio Model*.

**Figure 7:**
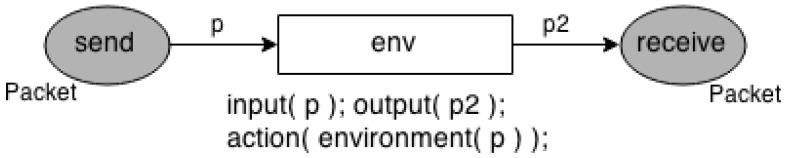
Environment Model.

The Environment Model is the propagation model, which is responsible for modelling everything in the middle between the sender and receiver sensor nodes. It identifies the connections between the sensor nodes and forwards the packets from a sensor node (source) to another (target). In Figure 7, places *send* and *receive* represent the time that a node sends and receives a packet, respectively. The transition *env* models everything between the sensor nodes, e.g., free space, indoor, outdoor.

### 4.3. Consumption Sensor Node Model

**Figure 8:**
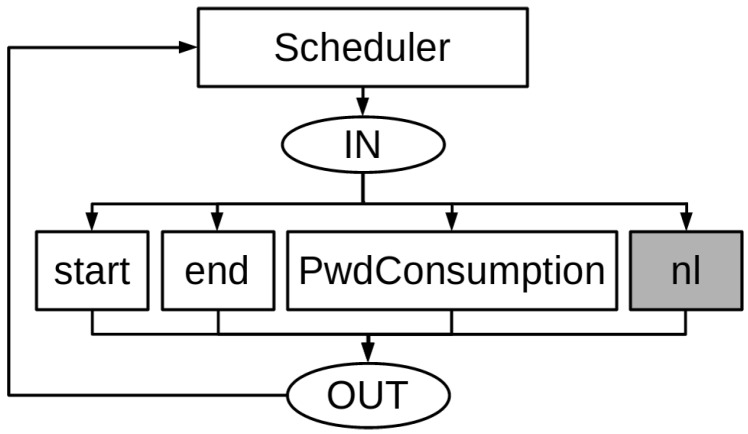
Consumption Simplified Model of the application.

**Figure 9:**
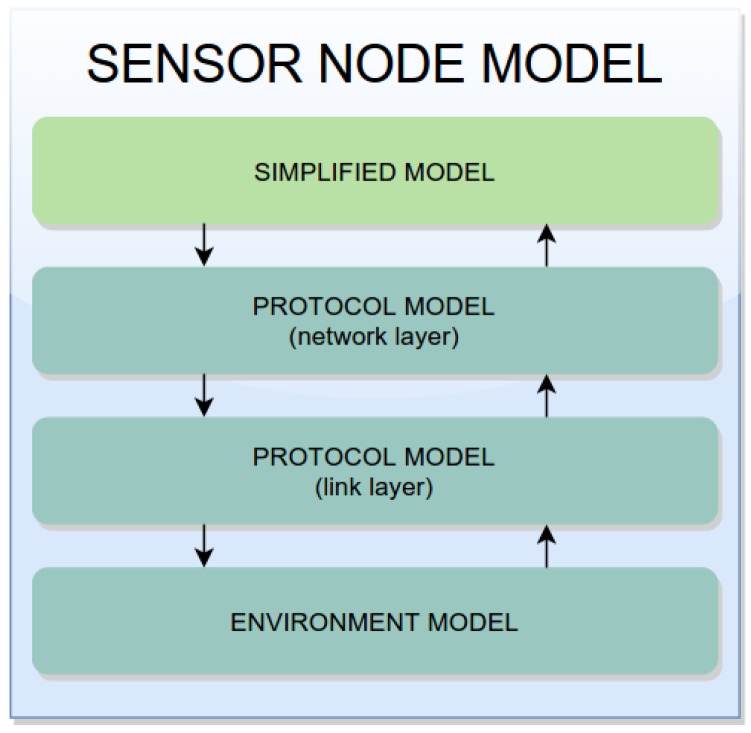
Overview of the Consumption Sensor Node Model.

The Consumption Sensor Node Model is responsible for evaluating the power consumption of the application and network together by combining their models (Consumption Application Model and Consumption Network Model) [16]. Due to their size and complexity, it is extremely costly to simulate this combination. Hence, it is necessary to evaluate the Consumption Application Model in separate and gets its power consumption and the packet transmission frequency. The Consumption Simplified Model (Figure 8) uses these values. Transition *PwrConsumption* models the power consumption of the entire application. In this way, the Sensor Node Model considers aspects of the application without changing its structure and behaviour (as can be seen in Figure 9).

### 4.4. Reliability Model

The Reliability Model [18] is used to assess the reliability of a WSN as defined in activity *Evaluate Reliability Models* of Figure 3. Similar to the previous models, there is a step-by-step to create it. The Reliability Model is made by path models that represent a path between a sensor node and the sink node; and small blocks (called basic blocks) that represent the sensor nodes or communication links. This model is created based on the results of the WSN power consumption assessment, as defined in the *Validation* phase of the methodology presented in Figure 3.

**Figure 10:**
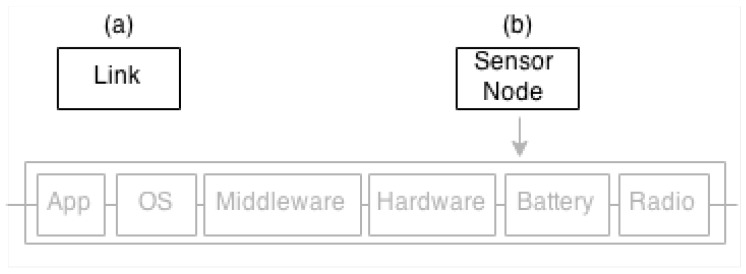
Basic blocks of the reliability model.

The failure points in WSNs are the communication link and the sensor node. For that reason, these two points are modelled as RBD blocks as shown in Figure 10.

In particular, the *Sensor Node* is a sequence of blocks, namely Application (*App*), Operating System (*OS*), Middleware (*Middleware*), Platform (*Hardware*), Radio (*Radio*) and Battery Level (*Battery*). If one of them fails, the block *Sensor Node* fails (see Section 2.2). Each block has a reliability associated, which is defined by the user (e.g., Application) or by simulation (e.g., Battery Level and Radio).

**Figure 11:**
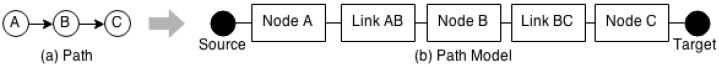
Path Model.

The reliability model of a path (called *Reliability Path Model*) consists of a sequence of basic blocks as shown in Figure 11. A path has at least two sensor nodes: one sensor node is the source (first block), and the other is the target (last block). If there are more sensor nodes (multihop), they are placed between the first and last blocks. Besides, it is worth observing that the *Reliability Path Model* depends on the routing algorithm adopted as it determines whether the path is single hop or multihop; and the sink node is always the target (last block).

For example, Figure 11a shows a path from the sensor nodes *A* (source) to *C* (target), in which sensor node *B* routes the packet between the source and the target. The model of this path is shown in Figure 11b: a model in series because the communication is sequential. If another sensor node routes a packet between the source and the destination (simultaneously), we need a parallel structure.

After modelling all paths, the next step is to model a region. A region includes one or more sensor nodes. If the region has only one sensor node, its path model is considered the Reliability Model. Otherwise, in the case the region includes more than one node, it is needed to combine the path models of these sensor nodes: the combination process checks if a particular sensor node belongs to different paths.

**Figure 12:**
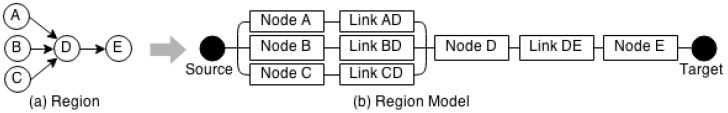
Reliability Model.

For example, Figure 12a illustrates a region and its paths to the sink node. This region uses a protocol that creates clusters (e.g., LEACH), has three sensor nodes (*A*, *B* and *C*) and has a cluster head (*D*). Thus, all sensor nodes in this region must transmit their packets to the cluster leader. This means that all paths have one sensor node in common and this situation should be represented in the Reliability Model (Figure 12b). If this node fails, the whole region fails regardless of the path.

In some specific scenarios, a region can not be modelled by a series-parallel composition in RBD. In such cases, the method SDP [19] should be applied to evaluate the reliability of the WSN region.

## 5. Tooling

The tooling EDEN, available at https://github.com/sensor2model-group (*Evaluation and Development Environment for wireless sensor Networks*) was designed and fully implemented to support the methodology presented in Section 3.

EDEN is composed by four main components, namely *Editor*, *Translator*, *Evaluator* and *Manager*. *Editor* is a Web application used to develop the nesC code and design the network. It has two key features that help developers to improve the power consumption of WSNs: it suggests improvements to the nesC code reduce the power consumption; and it allows the developer to analyse, among many WSN configurations, which is the best configuration of the WSN. *Translator* generates power consumption and reliability models from the application source code and the defined network design. *Evaluator* is responsible for assessing the models created by *Translator*. Finally, *Manager* controls the entire process of the WSN evaluation by coordinating the tools. In other words, the *Editor*, *Translator* and *Evaluator* do not communicate directly with each other. They communicate only with the *Manager*. In this way, this strategy makes the architecture scalable and distributed, allowing EDEN to support multiple instances of the same tool simultaneously.

**Figure 13:**
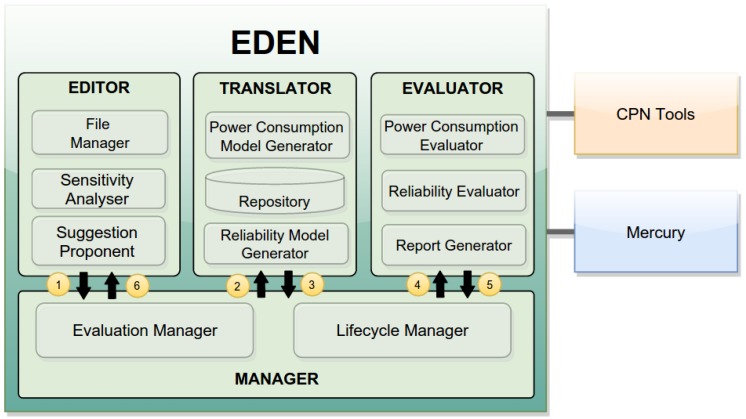
EDEN Architecture.

Figure 13 shows the architecture of EDEN. The *Editor* is composed by three components: *File Manger* is responsible for creating, reading, updating and deleting nesC applications; *Suggestion Proponent* gives suggestions on how to improve the power consumption of WSN applications (see Table 1); and *Sensitivity Analyser* is responsible for the sensitive analysis (see Section 2.3).

Regarding to *Sensitivity Analyser*, the developer must define which parameters (e.g., energy level, packet size) need to be evaluated. The network design is created and evaluated automatically, using the same sequence presented previously. After evaluating all network designs, the user decides which configuration has the best result (power consumption and/or reliability) according to the WSN planning. For example, the user may prefer that the WSN needs to have better power consumption than reliability.

The *Translator* has a repository (*Repository*) to storage the basic models (see Figure 3) and two components to create the CPN and RBD models, namely *Power Consumption Model Generator* e *Reliability Model Generator*.

The *Evaluator* has two components (*Power Consumption Evaluator* and *Reliability Evaluator*) to evaluate the models created by the *Translator*, and one component (*Report Generator*) to generate the reports with the simulation results. Finally, the *Manager* has the component *Evaluate Manager* to control the evaluation process; and *Lifecycle Manager*, to control the instances of the tools.

Figure 13 also shows a sequence of steps from the design to evaluation of the WSN. The developer only interacts with the *Editor* which is a Graphical User Interface (GUI). Through this interface, the user designs and creates the WSN applications and network design. While the user develops the WSN (application or network), the *Editor* proposes suggestions for improving the power consumption of the WSN.

After implementing the application and the network design, the user can evaluate the WSN power consumption and reliability using the *Manager* (Step 1). It receives the WSN project (application and/or network design) and forwards it to the *Translator* (Step 2), which converts the application and the network into CPN (power consumption) and RBD (reliability) models. When *Translator* concludes the translation process, it notifies *Manager* (Step 3) that receives the models created and forwards it to *Evaluator* (Step 4). Similarly, after concluding its task, *Evaluator* creates the reports and notifies *Manager* (Step 5). As illustrated in Figure 3, the application and the network are evaluated sequentially; as well as the power consumption and reliability. For that reason, *Manager* controls the sequence of evaluation of the models. If all models have been evaluated, *Manager* forwards the results to *Editor* (Step 6), which shows them to the user.

## 6. Experimental Evaluation

This section presents the experimental evaluation whose objectives can be summarised as follows: to show how the suggestions made at development time help to reduce the power consumption of WSN applications; to validate the proposed power consumption and reliability models; and to show how the sensitivity analysis can be used to select the best network configuration.

### 6.1. Suggestions

In order to show the power consumption reduction resulting from the suggestions proposed by the *Editor* (see Table 1), we carried out six experiments:

- Experiment 01 (*E01*): it evaluates the impact of the statement *for* (Suggestion *S01*) in the power consumption. This statement has three expressions: (1) the first one is associated with the initialization, (2) the second one is the controlling statement, which is assessed before the iteration, and (3) the last expression commonly defines the step size;
- Experiment 02 (*E02*): it evaluates statement *for* using increment (e.g., *for*(*i = 0 ; i < 10; i++*)) or decrement (e.g., *for*(*i = 10 ; i >= 0; i–*)) in the step size (Suggestion *S02*);
- Experiment 03 (*E03*): it evaluates the power consumption of the use or not of parenthesis (Suggestion *S03*);
- Experiment 04 (*E04*): it assesses the branch order of statement *if-then-else* (Suggestion *S04*), comparing the results obtained when the most accessible branch is the last or the first one;
- Experiment 05 (*E05*): it evaluates the three ways (var++, var+=1, var = var + 1) of incrementing a value in nesC (Suggestion *S05*); and
- Experiment 06 (*E06*): it checks if there is some difference in the application’s power consumption when it uses a variable, constant or fixed value (Suggestion *S06*).

These experiments were carried out by implementing six different nesC applications. The nesC applications were deployed in an IRIS mote, connected to a MTS400 basic environment sensor board. An oscilloscope was used to measure the power consumption of the mote where the applications execute. A computer attached to the oscilloscope obtains the code snippet execution start and end times through monitoring one of the LEDs of the mote, which is turned on/off to indicate the execution starting/ending. The PC runs a tool named AMALGHMA [20] that is responsible for calculating the power consumption. Each experiment was performed 100 times, and the confidence level is equal to 95%.

Two applications were used in Experiment *E01*. These applications initialize an array of size 20 and assign the value zero to all positions. First application uses the statement *for* to perform this task, while second code uses 20 individual assignments to carry out the same task.

**Figure 14:**
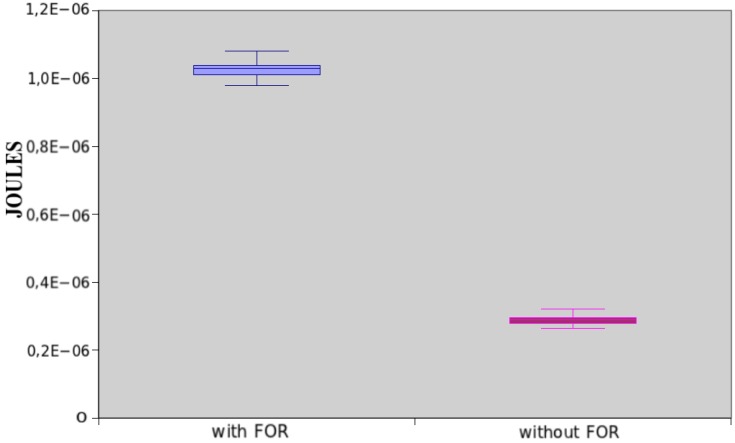
Power Consumption of E01.

The power consumption of both applications is shown in Figure 14. In this case, the first application (with statement *for*) consumes 71.85% more energy than the second one. We applied the hypothesis testing to the results, and it generated a *p*-value equal to $1.85816\times 10^{-65}$, which shows that statement *for* has a great impact on the power consumption of the application. This fact occurs because it has three expressions that consume a lot of energy: initialization of the value assignment, evaluation of a condition, and increase/decrease expression.

In Experiment *E02*, we implemented two applications: the first application uses an increasing step (from zero to twenty), and the second code uses a decreasing step (from twenty to zero). These applications initialize an array with size predefined equal to 20 and assign zero to all positions of the array.

In this case, the application that uses the decreasing step consumes 54.78% more energy than the one with increasing step. The hypothesis testing yielded a *p*-value equal to $7.7639\times 10^{-49}$. Hence, it shows that is better to use an increasing step solution. The possible reason for this difference may be an optimisation carried out by the processor or compiler, as the increment step is more common than the decrement one.

We measured the power consumption of two similar applications in Experiment *E03*. These applications calculate the average (*avg*) of five numbers (*a*, *b*, *c*, *d* and *e*) in two different ways: sum=(a+b+c+d+e) and avg=sum/5; and avg=(a+b+c+d+e)/5.

The mean power consumption of first application is 62.46% lower than the second one. By applying the hypothesis testing, it produces a *p*-value equal to $1.08015\times 10^{-53}$. As the confidence level is equal to 95% and the result is less than 0.05, it means that the two applications returned different values. Hence, we concluded that is better to use the first implementation, i.e., two assignments. To understand the reason for this difference, it is important to observe that the operator ’/’ has a higher priority of execution than instruction ’+’; and the parenthesis forced to change the priority in the execution order of the CPU instructions. When we used the parenthesis before operator ’/’, the CPU is forced to execute the operator ’+’ prior to ’/’. Hence, it consumed more energy to change the priority of the execution.

In Experiment *E04*, we implemented two applications that have one *if-then-else* statement with five branches and probability of occurrence associated with each branch. Four branches have 5% probability of occurrence, and one branch has 80%. In practice, if we execute this application twenty times, there is 80% of probability of occurrence of one of the branches. The difference between these applications is the order of branches. In the first code, the branch with probability 80% is the last to be checked. While in the second one, this branch is the first one to be evaluated.

The first application consumes 23.71% more energy than the second one. Similar others scenarios, we used the hypothesis testing, which returns $3.1065\times 10^{-44}$. This value means that the second application consumes less energy than the first code. The branch that executes more times should be evaluated as soon as possible.

In Experiment *E05*, we evaluated the three possibilities of incrementing a value (e.g., increment by 1) available in nesC: var++ (*Type01*), var += 1 (*Type02*) and var = var + 1 (*Type03*). We implemented an application for each of these possibilities.

*Type01* application has the lowest power consumption among the three implementations. However, as *Type02* application has the mean very close to *Type01*, we chose to investigate if those averages are significantly different. By using the hypothesis testing, it returned a *p*-value equal to 0.4632, which means that the power consumption of *Type01* and *Type02* applications cannot be differentiated.

However, *Type03* application consumes more energy than the two others: 20.82% and 19.83% more than *Type01* and *Type02*, respectively. Similarly to the previous experiment, we used the hypothesis testing that returned *p*-value equal to $5.5745\times 10^{-20}$ and $8.2415\times 10^{-28}$, respectively. These mean that the *Type03* is the worst implementation when an increment is necessary.

The last experiment, Experiment *E06*, evaluates a very common nesC statement: the assignment. Experiment *E06* assesses three variations of this command: assignment of a constant, assignment of a fixed value and assignment of another variable.

The power consumption of the constant and fixed value assignments are very similar, i.e., around 0.06% of the variation. As the values are very close, we used the hypothesis testing to verify whether this difference exists. The test produced a *p*-value equal to 0.9365, which means that these two ways returned similar values.

About the variable assignment, it has the highest power consumption: 23.31% higher than constant assignment and 23.27% higher that fixed value assignment. The hypothesis testing was used to ascertain whether these differences exist. It returned a *p*-value equal to $7.82489\times 10^{-33}$ and $4.7263\times 10^{-34}$ when compared with constant and fixed value assignment, respectively. This result means that in both cases the variable assignment consumed more energy.

It is worth observing that *Editor* (see Section 5) already implements the best power consumption strategies shown in these six experiments. Hence, WSN application developers are warned by the *Editor* when a better option (regarding power consumption) is not being used.

### 6.2. Models

To validate the CPN and RBD models presented in Section 4, three experiments were executed.

First experiment was carried out to validate the Consumption Application Model (see Section 4.1). In this experiment, we initially implemented a nesC application able to collect and send the temperature to another sensor node. By using EDEN, we generated the power consumption model of this application. Next, we directly measured the power consumption of the nesC application using an oscilloscope and compared the results against ones obtained by simulating the generated model.

**Figure 15:**
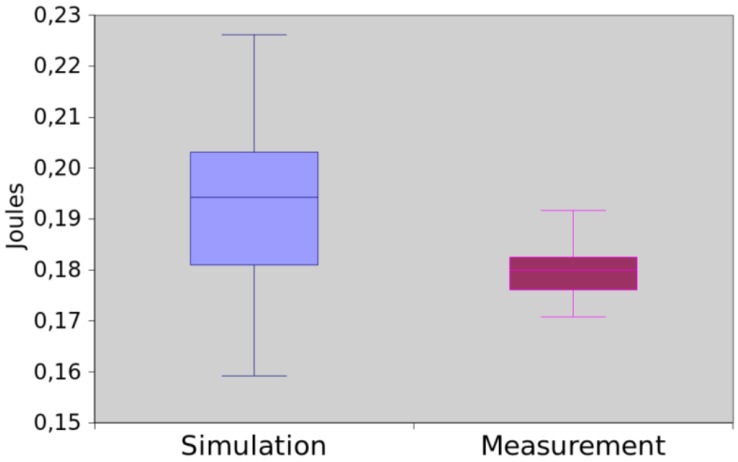
Power consumption of the application.

The results obtained by both methods are presented in Figure 15. The average difference between them was 6.71%. The hypothesis testing produced a *p*-value equal to 0.083238. As a consequence, for this experiment, we can assume that both methods return the same values.

In the second experiment, we replicated an experiment found in the literature to validate the Consumption Network Model. The analysis carried out by Senouci et al. [21] has a WSN with one sink node (at the centre of the WSN), 20 sensor nodes that send a packet every 2 s and the energy level is set to 1 mJ. Regarding the adopted routing protocols, we selected the protocols DIRECT, FLOODING, GOSSIPING and LEACH as they are widely documented and used in WSNs. Besides that, we chose four criteria of analysis: FND (when the first node is dead), 20% (20% of nodes are dead), HND (half of the nodes is dead) and LND (the last node is dead).

**Figure 16:**
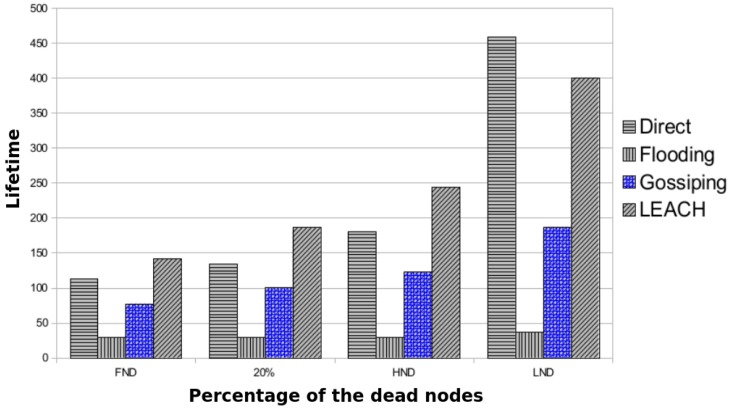
Power consumption of the network.

Figure 16 shows the percentage of the sensors nodes (*x*-axis) over the lifetime of the WSN (*y*-axis) using different protocols. As shown, the protocol FLOODING had the worst result, because it sends a packet in the broadcast. The protocol GOSSIPING had the second poorest result because it selects the next sensor node randomly. The protocol DIRECT and LEACH had the best results. Protocol DIRECT sends a packet directly to the sink node; however, the farthest nodes die earlier. Meanwhile, protocol LEACH creates clusters periodically in the WSN, balancing the power consumption among the sensor nodes. These results are comparable to ones obtained by Senouci et al. [21] except when half of the nodes are dead (HND), in which DIRECT had a better performance than the LEACH. More results about network aspects, such as network size and placement algorithms, were obtained in the sensitivity analysis as shown in Section 6.3.

The previous experiment was also used to assess the reliability of the WSN, more specifically, a region with one sensor node located at 100 m from the sink node. First, we evaluated the power consumption of the WSN and, next, we assessed the reliability of the WSN, because the energy level of the sensor node was used to calculate the reliability of them. The equation in the following was used to calculate the energy level:

$$R_{t}=\left(level_{t}-0.5394\right)/0.2788$$

where level_t is the energy level at time *t* and the values 0.5394 and 0.2788 were defined by [18]. Additionally, the link communication was considered perfect, and its reliability was set to 1.0.

**Figure 17:**
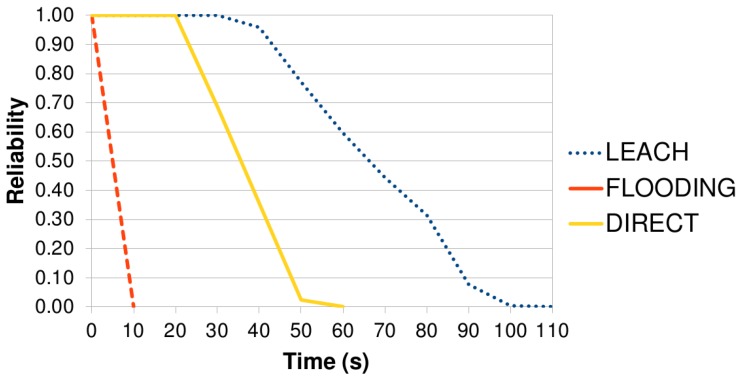
Results of the WSN reliability.

Figure 16 and Figure 17 show the WSN power consumption and the WSN reliability of the same experiment. As expected, the WSN reliability of the protocol FLOODING was the worst (see Figure 17) because the sensor node sends a packet in a broadcast. The protocol DIRECT had the second best result because the sensor node is far from the sink node and dies earlier. If the region was closer to the sink node, the protocol DIRECT could have the best WSN reliability. The protocol LEACH had the best result because it creates clusters: a sensor node (leader of the cluster) receives data from the other, aggregates the data and sends it to the sink node. As the leaders are elected from time to time, this mechanism helps to balance the power consumption.

Protocol GOSSIPING was not considered because its reliability has an atypical behaviour: its reliability does not decrease following a pattern over time. This fact happens because GOSSIPING randomly selects the sensor nodes to forward the packets, and the reliability is directly related to the energy level of the sensor node. For example, consider that protocol GOSSIPING selects nodes *X* and *Y* to forward packets at times $T_{0}$ and $T_{1}$, respectively. If node *Y* has more energy than node *X*, GOSSIPING has reliability at time $T_{1}$ greater than $T_{0}$. In other words, the reliability increases from $T_{0}$ to $T_{1}$. A worse scenario would be if node *X* has 0% of energy (reliability = 0) and node *Y* has 100% of energy (reliability = 1). The reliability of GOSSIPING would change from 0 to 1. This atypical behaviour makes difficult to compare its reliability with other protocols.

### 6.3. Sensitivity Analysis

Activity *Do Sensitivity Analysis* (see Figure 3) is responsible for creating and evaluating multiple WSN configurations according to the parameters (called factors) and values (called levels) defined by users. This activity is an essential one for identifying the influence of each factor on power consumption and reliability of WSN.

To perform the sensitivity analysis, we considered the factors and levels shown in Table 2. We chose these factors because they are widely known in WSN domain. About the levels, they will be explained in the next paragraphs.

We defined that the first level (−1) is the worst case and second one (+1) is the best based on the results of the WSN power consumption [16,18,21,22,23]. Positive numeric levels are five times higher than the next level of the same factor, e.g., 0.001 Joules and 0.005 Joules. In this way, we avoid favouring a particular factor to influence more or less the power consumption and/or reliability of the WSN.

The values assigned to *Initial Energy Level* (0.001 and 0.005) and *Node Number* (20 and 100) were defined based on ones adopted in [16,18,21]. Furthermore, it is worth observing that there is a relationship between the factors *Sending Regular Rate* and *Packet Size*. If a sensor node sends a 50 kb packet (first level of the Packet Size) every 2 s (first level of *Sending Regular Rate*), the same amount of data can be sent over the same period of time if it sends 250 kb every 10 s (second level of the *Packet Size* and *Sending Regular Rate*, respectively). The goal is to check if it is more advantageous to frequently send small packets or send a large packet in a longer period. Additionally, all sensor nodes send the packet to the sink node and the periodicity in seconds helps to accelerate the evaluation process because it only finishes when all sensor nodes die, i.e., the energy level is 0 Joules.

Another relationship is between the *Place Size* and *Initial Radio Range*. It is important to note that the sink node is located in the centre of the WSN. For example, if the *Place Size* is 500 m × 500 m, the sink node is in the location (250,250). To ensure that any sensor node has direct communication with the sink node, it is necessary that all sensor nodes have an *Initial Radio Range* of the (at least) 250 m, which is the first level of this factor.

The routing protocols called GOSSIPING and DIRECT were chosen because they are the worst and the best power consumption according to [16,21], respectively. In fact, the protocol FLOODING had the worst performance in the two mentioned papers. However, it was not used because the network did not spend much time active, making it impossible to evaluate the impact on the WSN reliability.

**Figure 18:**
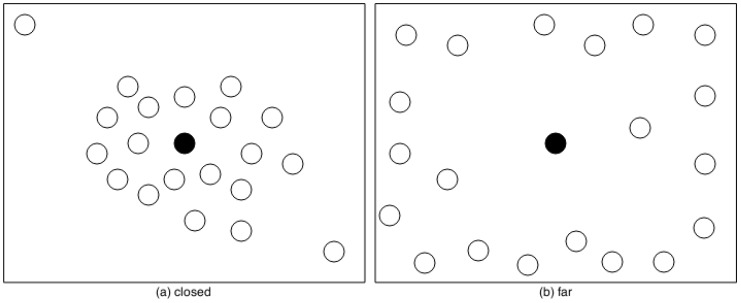
Sensor nodes are deployed (**a**) closed and (**b**) far from sink node.

Finally, the levels of *Placement Algorithm* are based on the Deployment Strategy-Oriented Lifetime [22,23]. The WSN can contain two regions (see Figure 18), one near and another far from the sink node, which is located in the centre of the WSN as mentioned before. The WSN power consumption will be different because sensor nodes need more energy to transmit a packet when they are far from the sink node. Besides that, the WSN reliability may vary according to the distance from the sensor node to the sink node, because WSN reliability is affected by the WSN power consumption as shown by the third experiment in Section 6.2.

It is worth observing that the factors *Routing Protocols* and *Placement Algorithm* are closely related. For example, the use of the DIRECT protocol is possible if all nodes are close enough to the sink node in such way that they can send and receive data to/from it. In this case, we could have considered *Routing Protocols* and *Placement Algorithm* as a single factor. However, we analysed them separately. Firstly, the implemented tooling (Section 5) allows evaluating different combinations of routing protocols and placement algorithms, e.g., the DIRECT protocol with nodes far from sink node. Secondly, the sensitivity analysis can show the interaction impact between these factors in a very clear way.

From these factors and levels, 256 combinations were created using Equation (3) and each combination is a WSN configuration. To carry out the sensitivity analysis, we used the component *Sensitivity Analyser* (see Figure 13) that can evaluate all combinations in parallel using multiple computers simultaneously.

#### 6.3.1. Considerations

Prior to present the results, it is important to understand some issues of the WSN simulation:

- First Order Radio Model [17,24] was used to calculate the power consumption of the WSN due to its simplicity. It only considers the power consumption when the sensor node sends and receives a packet, i.e., it is not examined the power consumption when the sensor node processes a packet, only when it sends or receives one;
- The communication between the sensor nodes is perfect and has no obstacles to degrade the signal. For example, if sensor node *A* is inside of the range communication of the sensor node *B*, sensor node *A* will receive the packets from sensor node *B*. Allied to this, it was also considered that the link layer is perfect, e.g., it avoids collision of packets. This is not an actual scenario, but it facilitates to understand which factors most affect power consumption and reliability because we have no external interference in the WSN power consumption and reliability (e.g., noise or packet collision);
- We defined a region (called *Region I*) with only one sensor node located at an edge of the WSN, specifically, between coordinates (0,0) and (10,10). This region is used to evaluate the impact of the factors on the WSN reliability. In fact, we already know that the distance (combination of the factors Place Size and Place Algorithm) and the routing protocol influence the reliability of a region [16]. However, we do not know which factor most affects the WSN reliability. Putting the region away from the sink node, we will have a better idea of which factors (besides those mentioned above) have an impact on the WSN reliability; and
- All sensor nodes send data to the sink node, which is located in the centre of the WSN. For example, the sink node will be located at the coordinate (50,50) and (250,250) when the *Place Size* is 100 m × 100 m and 500 m × 500 m, respectively.

#### 6.3.2. Results

We analysed the impact of each factor (e.g., *Routing Protocol*) and the interaction between two factors (e.g., *Routing Protocol and Packet Size*) on the WSN power consumption and reliability as shown Table 3. We obtained these values when we used Equations (4) and (5) in the results. In this table, the values are sorted in descending order, i.e., it begins with the factor or interaction with the greatest impact and ends with the lowest one.

*Power Consumption.* The four factors that affected more the WSN power consumption were *Routing Protocol* (RP), *Packet Size* (PaS), *Initial Energy Level* (IEL) and *Sending Regular Rate* (SRR). *Routing Protocol* (RP) has the greater impact among the four factors because it decides how the sensor nodes will communicate with the sink node. For example, the protocol DIRECT has a better power consumption than protocol GOSSIPING because sensor nodes send data directly to the sink node. It is worth observing that sensor nodes closer to the sink node are more active than sensor nodes far from it.

To understand the impact of *Packet Size* (PaS) and *Sending Regular Rate* (SRR), it is necessary to remember the use of the mentioned First Order Radio Model. It only considers the power consumption of sending and receiving packets; which is directly influenced by two factors: *Packet Size* (PS) and the *Initial Radio Range* (IRR). When we increment 5 times (e.g., 50 kb to 250 kb) the *Packet Size* (PaS), the sensor node will consume five times more to send a packet; and, when we change five times (e.g., 250 m to 1250 m) the value of the *Initial Radio Range* (IRR), the sensor node should consume 25 times more to send a packet (based on *First Order Radio Model*). However, this behaviour did not happen when we increment the *Initial Radio Range* (IRR), because the *Routing Protocol* (RP) changes it at runtime. For example, the protocol DIRECT changes (in all experiments) the value of the factor *Initial Radio Range* (IRR) to a value equal to the distance between the sensor node and the sink node. For that reason and because the sink node is in the centre of WSN, *Place Size* (PlS) influences more the WSN power consumption than *Initial Radio Range* (IRR).

Besides *Packet Size* (PS), *Sending Regular Rate* (SRR) also has a strong impact on the WSN power consumption. If the sensor node frequently sends several small packets, this behaviour increases its power consumption. The reason is straightforward: it consumes the same energy in the same period if it sends a big packet once or if it sends a small packet frequently. For example, two sensor nodes (*A* and *B*) send a different packet at different periods: the sensor node A sends a packet of 50 kb every 2 s; while the sensor node B sends every 250 kb every 10 s. Their power consumption will be the same every 10 s because they will send 250 kb at this period. However, *Packet Size* (PS) influences the WSN power consumption more than *Sending Regular Rate* (SRR) because it directly affects the WSN power consumption. For that reason, it is more important to reduce *Package Size* (PS) and increase *Sending Regular Rate* (SRR). Additionally, the interaction between *Packet Size* (PS) and *Sending Regular Rate* (SRR) is the 8th in the ranking (out of 36), which shows that there is a strong interaction between them.

The *Place Algorithm* (PA) also has a strong influence (less than the previous factors) on the WSN power consumption due to the selected routing protocols. The interaction between them is the 5th in the ranking. For example, as mentioned before, the protocol DIRECT benefits the sensor nodes close to the sink node.

Finally, *Node Number* (NN) has little impact on the WSN power consumption because of two factors: (i) protocol DIRECT does not require any other node to send the data to a sink node; and (ii) the link layer protocol was considered as perfect, i.e., it does not account for power consumption to receive packets by mistake or collision. If the experiment would use other protocols of the Link Layer and Routing Layer, the number of sensor nodes and the distribution could have a more significant impact on the WSN power consumption.

*Reliability.* WSN reliability was calculated at time 25 s because the most of the WSNs evaluated have the reliability greater than 0 at this time. Since the purpose of sensitivity analysis is to use the same factors and levels to assess the power consumption and reliability of WSNs, we evaluated the reliability of the *Region I* and we only considered battery level as the reliability of the sensor node (see Section 4.4). Without this consideration (battery level as reliability), the reliability of the sensitivity analysis would have to be done using other factors and levels (e.g., the reliability of the communication link).

The four factors that affected more the WSN reliability were *Routing Protocol* (RP), *Place Size* (PlS), *Packet Size* (PaS) and *Initial Energy Level* (IEL). As mentioned in [18], the WSN reliability varies according to the distance between the *Region I* and the sink node, the routing protocol used and the number of sensor nodes inside of the region. As the *Path Model* (see Section 4.4) is based on the routing algorithm, we expected that the routing protocol interferes on the WSN reliability. Besides that, as the region is located on the edge of the WSN (as mentioned in the Section 6.3.1) and the sink node in the centre of WSN, we also expected that the *Place Size* (PlS) is one of the factors that affected the WSN reliability. The *Node Number* (NN) did not affect so much the WSN reliability because the region had only one node independent from the number of sensor nodes of the WSN.

To understand the other factors, it is necessary to remember that the battery level was considered in the reliability of the sensor node. Thus, the factors that affected the WSN power consumption also affect the WSN reliability. Note that, from *Packet Size* (PaS), the order of impact was maintained. The only exception was *Placement Algorithm* (PA): it does not affect the WSN reliability as well as the WSN power consumption because the region was always in the same position regardless of the routing algorithm used. However, it is noteworthy that the interaction between *Placement Algorithm* (PA) and *Routing Protocol* (RP) had a median impact (11th position) in WSN reliability, because (most likely) *Routing Protocol* (RP) had a higher impact on the WSN reliability.

#### 6.3.3. Summary

The power consumption and reliability of WSNs were most affected by three factors in common: *Routing Protocol* (RP), *Packet Size* (PaS) and *Initial Energy Level* (IEL). It should be given preference to routing protocols that consumes very few energy and do not use many sensor nodes to route packets (better reliability); sensor nodes should send small packets; and (when possible) put a battery with a higher load of energy in sensor nodes. As a fourth factor, the WSN reliability requires attention to the position of the sink node; while, for its power consumption, the sensor nodes should send a packet when it is necessary, i.e., only when an event occurs.

## 7. Related Work

This section presents existing approaches on the evaluation of power consumption, reliability, and combined reliability and power consumption of WSNs. Regarding the large number of works that deal with general aspects of reliability and power consumption in WSNs, we concentrate on proposals that use formal models to specify and assess these quality attributes. Besides, as we adopted sensitivity analysis, we also include existing studies on the utilisation of this technique in WSNs.

### 7.1. Power Consumption

Different formal techniques have been used to model and evaluate the energy consumption of WSNs: Petri nets [25,26], queuing Petri net (QPN) [27], Colored Petri net [16,18], Markov chains [28,29], Finite State Machines [30,31], process algebras [32].

Shareef [25,26] used Petri nets to model processors of WSN nodes. By using the proposed models, he estimates the average CPU power consumption. Besides, the model is also used to understand the energy consumption at the network layer of the cross-layer energy-aware routing.

Li [27] presented an event-driven queuing Petri net (QPN) model to simulate the power consumption of WSN nodes. Each node has an energy source (battery) and a set of components, such as microcontroller, receiver and sensors. In turn, each component has its power states and some preset state transitions that define its power behaviour, which is modelled in QPN. While the power evaluation is based on QPN, reliability issues are not considered in this evaluation.

Damaso [16,18] proposed the use of Coloured Petri nets to evaluate the power consumption of both the sensor network lifetime and WSN application executing in the node. This approach consists of a fully automated process for assessing the WSN lifetime, a set of reusable Coloured Petri Net models to express the power consumption of WSN communication protocols, and a strategy for comprising CPN models of WSN applications and protocols.

Wang [28] presented a Markov chain-based cross-layer model to study the statistics of power consumption of WSNs. In particular, this work captures the probabilistic distribution of the energy consumption. In practice, instead of the average power consumption, this approach produces the probability that the consumed power in a given period is lower than a particular threshold.

Bruneo [29] designed two battery models (linear and non-linear) to perform a parametric evaluation of the longevity of WSN nodes. The linear model is based on a Markov reward model that allows capturing the battery consumption of the WSN node and determining some reliability parameters. Meanwhile, the non-linear model is based on continuous phase-type distributions and Kronecker algebra.

Kelner [30] defined a power consumption model based on finite state machines. In particular, the model is used for an online account, simulation and generation of a prior knowledge of the WSN. The proposed model consists of FSMs that defines states and transitions of WSN nodes’ hardware, in such way that moving through the model and summing up annotated energy values enable us to estimate the power consumption of the node.

Snadjer [31] designed FSMs (Finite State Machines) to model the behaviour and interactions of WSN node elements, namely processing, communication and control units. In particular, the emphasis is on the communication unit where every transition of the FSM alters the energy consumption of the node. The measurements of the actual consumption of ZigBee and Wi-Fi communications feed their respective FSMs.

Gallina [32] proposed a framework for modelling WSNs that can be used to evaluate their power consumption. The framework is based on a probabilistic process algebra whose functioning is driven by Markovian probabilistic schedulers. The use of a process algebra allows checking temporal logic properties using model checkers. Each WSN node along with its location is represented in such way that probability distributions drive the node mobility.

More recently, Elhoseny [33] adopted a Genetic Algorithm (GA) in a solution to monitor targets in WSNs. GA is used to optimise the monitoring coverage of non-stationary sensor nodes and to maximise the network lifetime. The proposed algorithm utilises information, such as the coverage range of individual sensor nodes, distance to base station and expected consumed energy, to choose the sensor cover that minimises the power consumption to monitor the targets.

Similarly to what is being proposed in this paper, these works also use formal techniques or formal approaches to evaluate the power consumption of several aspects of WSNs, e.g., applications, nodes and communication protocols. Whatever the approach, however, the reliability of the WSN is not considered in these related works.

Finally, it is worth mentioning that no comparisons between existing energy math models (associated with the network power consumption) were necessary due to two main reasons: no new energy math models have been proposed, and the network model is parameterised in such way that it can use any energy math model. As mentioned in Section 4, the proposed network model already uses a simple and widely used energy model [17].

### 7.2. Reliability

Existing works on assessing the reliability of WSNs have adopted RBD [34], DRBD [35], Markov Chain [36,37], HOL [38], minimal path set [39].

Damaso [34] proposed the use of RBD for evaluating the reliability of WSNs. The reliability of the whole WSN is defined regarding the reliability of nodes and communication links. In turn, the reliability of the node is calculated considering the applications executing on the node, operating system, middleware, hardware, radio and. The reliability of the links is influenced by the routing algorithms used in WSNs.

Distefano [35] proposed the use of DRDB (Dynamic Reliability Block Diagram) and Petri to evaluate the reliability of WSNs. A WSN is viewed as a system made of nodes and whose reliability is defined regarding the reliability of individual nodes. Besides, due to sleep/wake-up standby policies and possible interference of the wireless communication, the WSN is viewed as a dynamic system.

Vasar [36] used Markov models to evaluate the reliability of WSNs. These models assume that a WSN node has a redundant replacement node that can replace the faulty one without delay (hot-standby).

Shen [37] addressed reliability of WSNs in a particular scenario having clustered sensor networks under epidemic-malware propagation. The authors adopt a strategic game to predict malware’s infection behaviour. Next, they integrate the behaviour into the transition probability upon infection of a WSN node. From this point, it is possible to determine how to relate the intent of malware infection to the CTMC’s (Continuous-Time Markov Chain) randomness. Finally, Shen proposes a novel measure to reflect the reliability of a WSN node.

Ahmad [38] adopted HOL (High-Order Logic) theorem prover to analyse RBD-based models of WSN transport protocols. In practice, Ahmad uses HOL to formalise series, parallel and parallel-series RBDs, which are then used to analyse the reliability of the end-to-end (e2e) data transport mechanism, and the Event to Sink Reliable Transport (ESRT) and Reliable Multi-Segment Transport (RMST) data transport protocols.

Mejjaouli [39] used minimal path sets to model the reliability of WSNs. The path set is defined as the minimal set of functioning WSN nodes that ensure a certain threshold of events detected by the sensor network. The proposed models are then evaluated using a discrete-event simulation environment.

Zhu [40] presented models to evaluate the reliability of WSN transmissions.The proposed models are mission-oriented and based on transmission paths. The models are defined mathematically and evaluated by a dynamic framework also proposed by the authors.

These mentioned approaches concentrate on the analysis of reliability separately, i.e., they do not consider the impact of the power consumption on the reliability. Additionally, the sensitivity analysis proposed in this paper helps to deal with the multitude of parameters when a WSN configuration needs to be selected.

### 7.3. Combined Power Consumption and Reliability

The strategy proposed by Zonouz et al. [4] to evaluate the power consumption and reliability of WSNs is very similar to one raised in this paper. They used graphs with mathematical expressions (e.g., First Order Radio Model) to represent and to evaluate the power consumption and the reliability of the WSN. They consider the battery level as reliability factor for the link communication, while we consider as the reliability of the sensor node. Furthermore, they evaluated the power consumption and reliability of the WSN at the same time when simulates the graph. While we evaluated sequentially: first, power consumption (CPN models) and, then, reliability (RBD models) using different models. Besides that, we define the power consumption of the application through its source code; while they define a constant power consumption of the application. Unlike Zonouz, the whole process is guided by a methodology and supported by a tooling.

### 7.4. Sensitivity Analysis

The use of sensitivity analysis in WSNs evaluates the factors that impact on the location algorithms (identifying the position of the sensor nodes) [41,42] and the routing protocol.

Sahota [41] studied the WSN for agricultural precision. He focuses on the protocol stack (link and physical layers) and shows the importance of the location algorithms (identifying the position of the sensor nodes) for this type of application. In particular, he used the sensitivity analysis to determine the amount of error (output) according to the parameters utilised by the location models (input). In particular, it defines that the location based on the arrival time is more precise than one based on the received signal strength.

Similarly, Yan Ma and Ma Jin [42] evaluated the factors that influence the location in a variant of WSN to the aquatic environment (Underwater Wireless Sensor Array Networks—UWSAN). A UWSAN has many sensor nodes equipped with an array of sensors to collect and process electromagnetic or acoustic signals. In this case, acoustic signals are used by location algorithms to determine the position of the sensor nodes. More specifically, they studied a relation between the Direction of Arrival (DoA), that is angular of reception, to the location based on the Least-Square Method. Among several findings, they define that the error of the source location and the distance between the source and the sensor node increase when the number of the sensor nodes and DoA (angular of reception) are keeping.

Different from previous papers, our work does the sensitivity analysis of the WSN, while they evaluated variants of the WSN; and we show the impact of the each parameter on the power consumption and reliability of the WSN, while they demonstrate the influence on the location algorithms.

Our work is close to [43]: both perform the sensitivity analysis of the WSN and showed the impact of the parameters on the WSN power consumption. However, they are different in many aspects. For example, he evaluated the power consumption of the WSN using NS-2 and we used formal models. He considered that sensor node could move and used the radio propagation model as a parameter, while we studied a static network and only one radio propagation model. On the other hand, we considered the routing protocol as a parameter and evaluated the impact of each factor on power consumption and reliability of WSN. Besides that, we proposed a methodology for development and evaluation a WSN that is fully supported by the implemented tooling.

## 8. Conclusions and Future Work

This paper presented a solution to evaluate the power consumption and reliability of WSNs in an integrated way. The proposed solution consists of a methodology, a set of formal models and a tooling that uses the proposed models and automatize the methodology’s activities. The assessment of the proposed solution included an experimental evaluation to validate the proposed models, to show how the sensitivity analysis helps to select the best WSN configuration and to evaluate the impact of the suggestions on the power consumption analysis.

The main contribution of this paper is the integrated analysis of power consumption and reliability of WSNs. The integration was only possible due to the definition of a methodology that guides the modelling steps and the tooling that support all steps of the methodology. Meanwhile, as the entire methodology is based on CPN and RBD models specially designed to express power consumption and reliability, the set of proposed models is another key contribution of the paper. Finally, it is worth observing that the use of sensitivity analysis to help in designing WSNs is also a novelty.

Next steps are to add new activities in the methodology and enrich the models. For example, activities to evaluate safety and cost of WSNs, and activities to monitor the network after its deployment. Such changes in the methodology will reflect in the set of models and tooling, which should create and evaluate the models automatically, and send and receive information from WSN monitoring. About the models, we intend to enrich the set of models with additional communication protocols (e.g., routing protocols) and to consider the link and transport protocols to build the reliability model.

## Figures and Tables

**Table 1:** Implementation suggestions of *Suggestion Component*.

Suggestion	Description
*S01*	Avoid to use the *for* statement to initialise an array.
*S02*	Use the increment instead of decrement in the *for* statement.
*S03*	Avoid the use of parenthesis in mathematical expressions.
*S04*	Place most accessed branches of decision commands as first options.
*S05*	Use ++ instead of +1.
*S06*	If the value of the variable does not change, make it a constant.

**Table 2:** Factors and Levels.

Factor	Acronym	Level (−1)	Level (+1)
Energy Level	IEL	0.001 Joules	0.005 Joules
Packet Size	PaS	250 kb	50 kb
Routing Protocol	RP	GOSSIPING	DIRECT
Initial Radio Range	IRR	1770 m	354 m
Placement Algorithm	PA	Far	Closed
Node Number	NN	20	100
Sending Regular Rate	SRR	2 s	10 s
Place Size	PlS	500 m × 500 m	100 m × 100 m

**Table 3:** Impact of factors on the power consumption and reliability of the WSN.

	Power Consumption		Reliability	
	Factor	Impact	Factor	Impact
1	RP	2169.39844	RP	0.40058532
2	RP and PaS	−2021.21875	PlS and RP	−0.36882255
3	RP and IEL	−1992.00781	PlS	0.33705977
4	RP and SRR	−1970.54688	RP and PaS	−0.27112969
5	PA and RP	−1886.37500	RP and IEL	−0.27058203
6	PaS	1873.03906	RP and SRR	−0.26114040
7	PaS and IEL	−1843.82813	PlS and PaS	−0.23936691
8	PaS and SRR	−1822.36719	PlS and IEL	−0.23881926
9	IEL	1814.61719	PlS and SRR	−0.22937762
10	IEL and SRR	−1793.15625	IRR and RP	−0.20832578
11	SRR	1771.69531	PA and RP	−0.19865220
12	PA and PaS	−1738.19531	NN and RP	−0.19406566
13	PA and IEL	−1708.98438	IRR and PlS	−0.17656301
14	PA and SRR	−1687.52344	PA and PlS	−0.16688943
15	PA	1603.35156	PlS and NN	−0.16230288
16	PlS and RP	−1459.57813	PaS	0.14167405
17	PlS and PaS	−1311.39844	PaS and IEL	−0.14112640
18	PlS and IEL	−1282.18750	IEL	0.14057874
19	PlS and SRR	−1260.72656	PaS and SRR	−0.13168476
20	IRR and RP	−1241.35156	IEL and SRR	−0.13113711
21	NN and RP	−1210.56250	SRR	0.12169547
22	PA and PlS	−1176.55469	IRR and PaS	−0.07887015
23	IRR and PaS	−1093.17188	IRR and IEL	−0.07832249
24	IRR and IEL	−1063.96094	PA and PaS	−0.06919657
25	NN and PaS	−1062.38281	IRR and SRR	−0.06888086
26	IRR and SRR	−1042.50000	PA and IEL	−0.06864891
27	NN and IEL	−1033.17188	NN and PaS	−0.06461003
28	NN and SRR	−1011.71094	NN and IEL	−0.06406237
29	PA and IRR	−958.32813	PA and SRR	−0.05920728
30	PA and NN	−927.53906	NN and SRR	−0.05462074
31	PlS	749.75781	IRR	0.01606624
32	IRR and PlS	−531.53125	NN	−0.01245400
33	PlS and NN	−500.74219	PA and NN	0.00786746
34	IRR	313.30469	PA and IRR	−0.00639266
35	IRR and NN	−282.51563	PA	−0.00328091
36	NN	251.72656	IRR and NN	−0.00180612
